# Multi Platforms Strategies and Metabolomics Approaches for the Investigation of Comprehensive Metabolite Profile in Dogs with *Babesia canis* Infection

**DOI:** 10.3390/ijms23031575

**Published:** 2022-01-29

**Authors:** Ivana Rubić, Richard Burchmore, Stefan Weidt, Clement Regnault, Josipa Kuleš, Renata Barić Rafaj, Tomislav Mašek, Anita Horvatić, Martina Crnogaj, Peter David Eckersall, Predrag Novak, Vladimir Mrljak

**Affiliations:** 1 Laboratory of Proteomics, Clinic for Internal Diseases, Faculty of Veterinary Medicine, University of Zagreb, 10 000 Zagreb, Croatia; irubic@vef.hr (I.R.); jkules@vef.hr (J.K.); 2 Glasgow Polyomics, Wolfson Wohl Cancer Research Centre, University of Glasgow, Glasgow G61 1QH, UK; Richard.Burchmore@glasgow.ac.uk (R.B.); Stefan.Weidt@glasgow.ac.uk (S.W.); Clement.Regnault@glasgow.ac.uk (C.R.); 3 Department of Chemistry and Biochemistry, Faculty of Veterinary Medicine, University of Zagreb, 10 000 Zagreb, Croatia; rrafaj@vef.unizg.hr; 4 Department of Nutrition and Dietetics of Domestic Animals, Faculty of Veterinary Medicine, University of Zagreb, 10 000 Zagreb, Croatia; tmasek@vef.unizg.hr; 5 Department of Chemistry and Biochemistry, Faculty of Food Technology and Biotechnology, University of Zagreb, 10 000 Zagreb, Croatia; ahorvatic@pbf.hr; 6 Clinic for Internal Diseases, Faculty of Veterinary Medicine, University of Zagreb, 10 000 Zagreb, Croatia; martina.crnogaj@vef.hr; 7 Institute of Biodiversity, Animal Health and Comparative Medicine, College of Medical, Veterinary and Life Sciences, University of Glasgow, Glasgow G61 1QH, UK; David.Eckersall@glasgow.ac.uk; 8 Department of Chemistry, Faculty of Science, University of Zagreb, 10 000 Zagreb, Croatia; pnovak@chem.pmf.hr

**Keywords:** metabolomics, serum, *Babesia canis*, chromatography, mass spectrometry

## Abstract

Canine babesiosis is an important tick-borne disease worldwide, caused by parasites of the *Babesia* genus. Although the disease process primarily affects erythrocytes, it may also have multisystemic consequences. The goal of this study was to explore and characterize the serum metabolome, by identifying potential metabolites and metabolic pathways in dogs naturally infected with *Babesia canis* using liquid and gas chromatography coupled to mass spectrometry. The study included 12 dogs naturally infected with *B. canis* and 12 healthy dogs. By combining three different analytical platforms using untargeted and targeted approaches, 295 metabolites were detected. The untargeted ultra-high performance liquid chromatography-tandem mass spectrometry (UHPLC-MS/MS) metabolomics approach identified 64 metabolites, the targeted UHPLC-MS/MS metabolomics approach identified 205 metabolites, and the GC-MS metabolomics approach identified 26 metabolites. Biological functions of differentially abundant metabolites indicate the involvement of various pathways in canine babesiosis including the following: glutathione metabolism; alanine, aspartate, and glutamate metabolism; glyoxylate and dicarboxylate metabolism; cysteine and methionine metabolism; and phenylalanine, tyrosine, and tryptophan biosynthesis. This study confirmed that host–pathogen interactions could be studied by metabolomics to assess chemical changes in the host, such that the differences in serum metabolome between dogs with *B. canis* infection and healthy dogs can be detected with liquid chromatography-mass spectrometry (LC-MS) and gas chromatography-mass spectrometry (GC-MS) methods. Our study provides novel insight into pathophysiological mechanisms of *B. canis* infection.

## 1. Introduction

Canine babesiosis is a tick-borne disease of worldwide importance caused by intraerythrocytic protozoa of different *Babesia* species [1]. Babesiosis in dogs is caused by both large and small forms of *Babesia* (*B. canis*, *B. vogeli*, *B. gibsoni*, and *B. microti*-like isolates) [2]. The large *Babesia*, previously considered to be *B. canis*, is currently split into three distinct species, namely *B. canis*, *B. rossi*, and *B. vogeli* [2]. Canine babesiosis caused by *B. canis* is the most common infection of dogs in certain regions of Europe [3]. The occurrence of canine babesiosis is associated with clinical cases, mostly in spring and autumn, because the relatively mild and wet weather is ideal for ticks [4].

The disease can be clinically classified into uncomplicated and complicated forms. An uncomplicated form of babesiosis has been suggested to be a consequence of anemia caused by hemolysis [5] with symptoms of fever, anorexia, pale mucous membranes, splenomegaly, depression, and water hammer pulse [6]. The clinical manifestations of the complicated form are variable and related to the particular complications that develop, but most often, they are a consequence of the development of an excessive inflammatory response called ‘systemic inflammatory response syndrome’, or SIRS [7], and multiple organ dysfunction syndrome, or MODS [5,8]. Research investigations have confirmed that both the uncomplicated and complicated forms of *B. canis* infection are connected with host inflammatory responses [9,10], namely, the host immune response to the parasite dominates in the pathogenesis of babesiosis and causes a generalized uncontrolled inflammatory response of the host with the marked release of inflammatory mediators [11]. The main feature of the acute-phase response is the release of inflammatory mediators, such as eicosanoids and cytokines, which play a key role in the pathophysiology of SIRS [12]. Eicosanoids are lipid mediators produced by oxidation of arachidonic acid and regulate immunological responses and inflammatory reactions, while cytokines are beneficial for the host defense, but in the case of excessive production, they can cause tissue injury and organ damage. Generally, babesiosis is an important disease due to its great economic importance in the world, but also due to the fact that babesiosis and malaria have many similarities, in diagnosis, immune phenomena, and inflammatory mechanisms [13]. The emergence of innovative-omics technology, such as metabolomics, allows for strategies to identify altered metabolites among thousands of small molecules present in biological fluids and tissues [14]. Metabolomics methodologies have been divided into two distinct approaches: untargeted metabolomics and targeted metabolomics [15,16]. Untargeted metabolomics provides an overview of the entire metabolome present in cells, biofluids, or tissues, and focuses on the detection and relative quantification of small molecules in a sample, including unknown chemicals. Moreover, targeted metabolomics identifies and quantifies the abundance of defined groups of known, chemically characterized, and biochemically annotated metabolites.

A comprehensive investigation of a wide range of metabolites demands multiple analytical methods and platforms. Although liquid chromatography-mass spectrometry (LC-MS) is the most popular technique for metabolomics applications due to the high sensitivity, repeatability, and reproducibility [17,18], and is the more appropriate method to detect and identify lipids, the gas chromatography-mass spectrometry (GC-MS) approach provides better metabolite separation of amino acids, fatty acids, and carbohydrates, and generally avoids ion suppression, which is a major challenge in LC-MS experiments [19]. Therefore, in the present study, we applied an integrated metabolomics approach using LC-MS-based untargeted and targeted metabolomics and GC-MS-based metabolomics.

Previous research demonstrated that canine serum is a suitable biofluid for metabolomics studies because of a wide range of applications in different research areas such as cancer, intestinal dysbiosis, diabetes mellitus, and others [20,21,22]. Thus, due to the similarities to human patients, metabolomics investigations in dogs can help the study of human diseases and also improve veterinary therapy and diagnostics [23,24,25,26]. Although metabolomics study in dogs in veterinary research is a new field and provides an increasing interest, it is still in its infancy compared with investigations in the human medicine field [27]. Our previous study assessed the urine metabolic profile in dogs with babesiosis using untargeted and targeted MS-based metabolomics approaches, and demonstrated that kidney dysfunction accompanying canine babesiosis was associated with changes in amino acid metabolism, energy metabolism, and fatty acid metabolism [28].

It is known that babesiosis is a protozoan disease; thus, the application of metabolomics investigation in parasitology can be a promising aspect for deepening the understanding of parasite metabolism and host–parasite interactions [29]. Therefore, previous metabolomics analysis has been performed on *Leishmania* [30,31], *Trypanosoma* [32,33], and *Plasmodium* [34,35].

This study aimed to investigate metabolite profiles and possible changes in the serum metabolome between dogs naturally infected with *B. canis* and healthy dogs. The study also aimed to investigate novel metabolites associated with clinicopathological abnormalities in babesiosis and provide deep insights into *B. canis* infection. This provides a better understanding of metabolic pathways altered in babesiosis and can lead to a more precise and adequate diagnosis, prognosis, and monitoring of this disease in the future.

## 2. Results

### 2.1. The Metabolomics Dataset in Dogs Infected with B. canis

**Figure 1:**
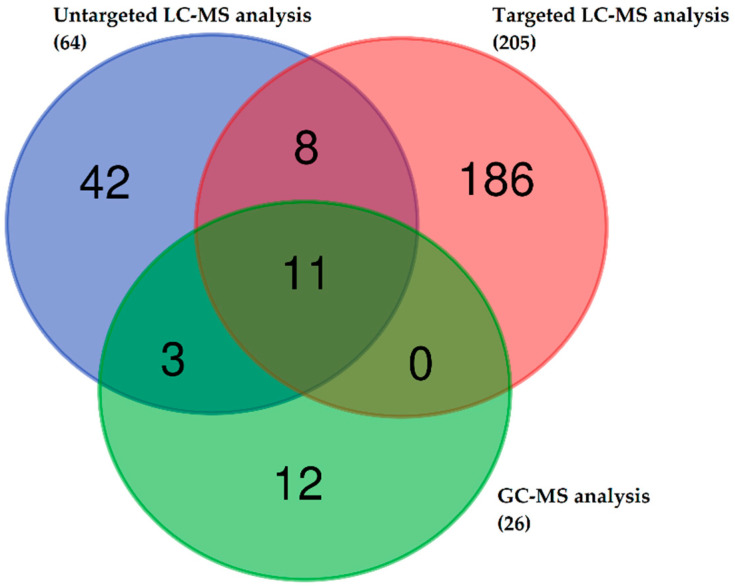
Venn diagram showing the number of metabolites identified in serum samples of dogs infected with *Babesia canis* and healthy dogs. The untargeted liquid chromatography-mass spectrometry (LC-MS) metabolomics approach identified 64 metabolites, the targeted LC-MS metabolomics approach (Biocrates analysis) identified 205 metabolites, and 26 metabolites were identified by gas chromatography-mass spectrometry (GC-MS) metabolomics approach. A total of 295 metabolites were detected using all three platforms.

In the final dataset, 295 different values of metabolite concentrations were identified for analysis (Figure 1). A total of 22 metabolites were identified using more than one platform: 14 metabolites were measured by LC-MS and GC-MS, 11 metabolites using Biocrates and the GC-MS platform, and 19 metabolites using Biocrates and the LC-MS platform including 11 metabolites that were measured using all three platforms. In this way, a total of 262 unique metabolites were quantified using at least one platform.

### 2.2. Untargeted LC-MS Metabolomics Analysis

An untargeted metabolomics approach was used to gain insight into the metabolome of dogs infected with *B. canis* and identify metabolites correlated with babesiosis. The metabolomics analysis resulted in detecting 1802 features from all 24 analyzed samples (Table S1A). A total of 64 metabolites were matched to known standards, and another 4098 compounds were annotated in the Polyomics integrated Metabolomics Pipeline (PiMP) using available databases. According to the *p*-value of <0.05, a total of 147 metabolic features were significantly changed (Table S1B). Among them, 14 metabolites were identified using standards (Table 1, Table S1C).

**Figure 2:**
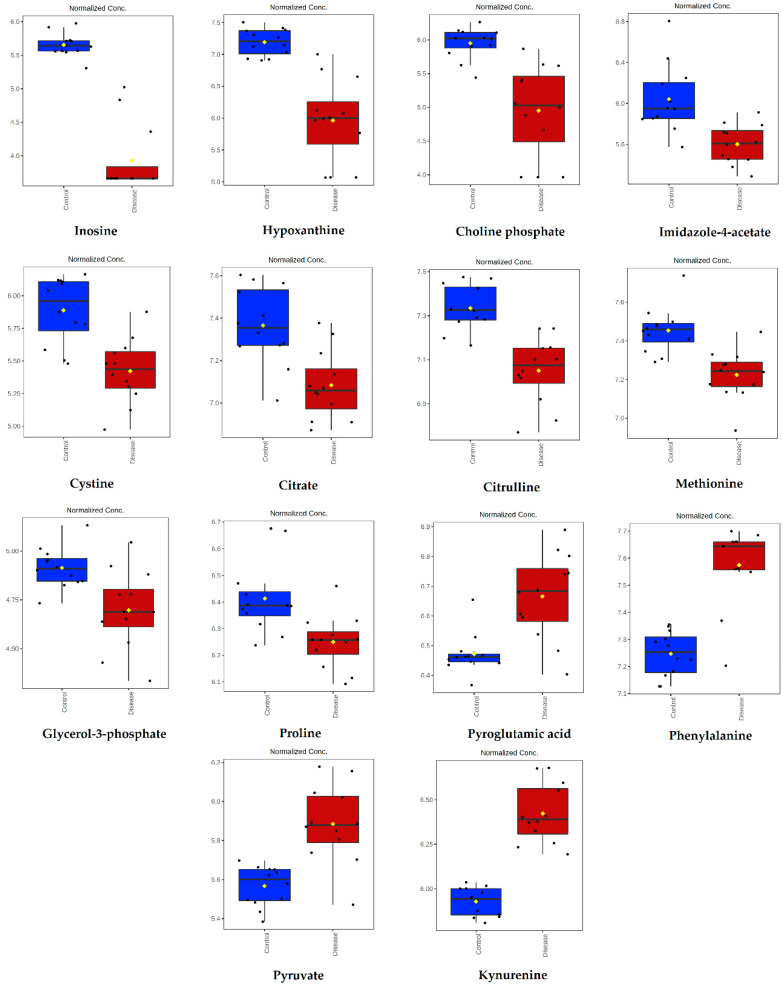
Intensity of metabolites significantly altered in dogs infected with *B. canis* and analyzed by the untargeted LC-MS metabolomics approach. Data are presented as box and whiskers plot (mean ± SD). All metabolites shown demonstrated a statistically significant difference between groups by *t*-test (*p* < 0.05). Blue-control samples, red-disease samples.

Inosine, hypoxanthine, choline phosphate, imidazole-4-acetate, cysteine, citrate, citrulline, methionine, glycerol-3-phosphate, and proline were lower in abundance, while pyroglutamic acid, phenylalanine, pyruvate, and kynurenine were higher in abundance in dogs infected with *B. canis* when compared to the control group (Figure 2).

### 2.3. Targeted LC-MS Metabolomics Analysis (Biocrates Analysis)

Targeted metabolomics analysis of serum samples was performed for 24 dogs using the Absolute IDQ-p400 kit (Biocrates Life Science AG, Innsbruck, Austria) through combined LC-MS and flow injection-mass spectrometry (FIA-MS) analysis. The datasets of 205 metabolites were used for statistical analysis and were classified into several groups, including amino acids (19), biogenic amines (8), monosaccharides (1), acylcarnitines (3), cholesteryl esters (9), diglycerides (9), triglycerides (31), lysophosphatidylcholines (12), phosphatidylcholines (88), and sphingomyelins (25). A total of 28 metabolites were absolutely quantified, 2 were quantified with restriction, and 175 were relatively quantified (Table S2A).

The univariate analysis resulted in the detection of 68 significant metabolites in healthy dogs versus dogs with babesiosis. A total of 10 significant metabolites were identified by LC-MS analysis. Among them, six were amino acids, and the rest were biogenic amines (Table 2).

A total of 58 significant metabolites were determined by FIA-MS analysis (Table S2B). The metabolites were classified into groups of lysophosphatidylcholines (5), diglycerides (7), phosphatidylcholines (25), triglycerides (11), sphingomyelins (8), and cholesteryl esters (2). The five most significant metabolites were as follows: lysophosphatidylcholine 22:6 (LPC (22:6)), diacylglycerol 36:2 (DG (36:2)), phosphatidylcholine O-38:6 (PC-O (38:6)), phosphatidylcholine O-40:6 (PC-O (40:6)), and diacylglycerol 36:3 (DG (36:3)).

**Figure 3:**
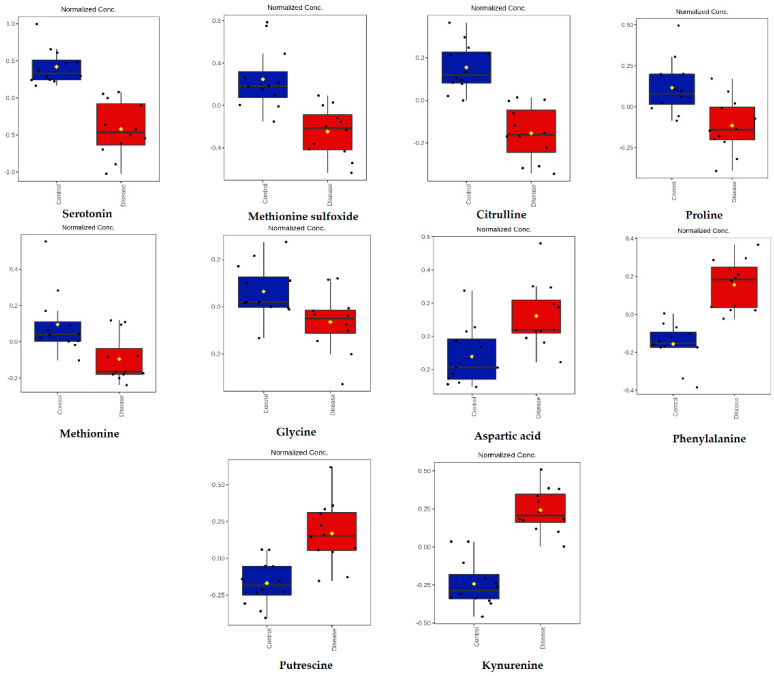
Concentrations of metabolites significantly altered in dogs infected with *B. canis* determined by the targeted LC-MS metabolomics approach. Data are presented as box and whiskers plot (mean ± SD). All metabolites shown demonstrated a statistically significant difference between groups by *t*-test (*p* < 0.05). Blue-control samples, red-disease samples.

Serotonin, methionine sulfoxide, citrulline, proline, methionine, and glycine were lower, while aspartic acid, phenylalanine, putrescine, and kynurenine were higher in dogs infected with *B. canis* (Figure 3).

### 2.4. GC-MS Metabolomics Analysis

The GC-MS-based metabolomics analysis was performed using 24 serum samples of dogs. A total of 26 metabolites were identified using GC-MS Metabolite Mass Spectral Database (Shimadzu, Kyoto, Japan). Most identified metabolites were classified as amino acids, while the other groups included organic acids, carbohydrates, fatty acids, and sugar alcohols (Table 3).

**Figure 4:**
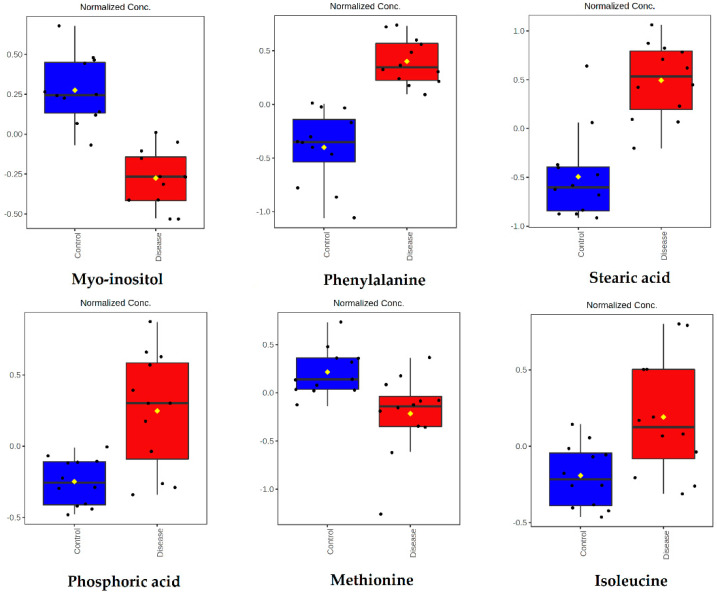
Concentrations of metabolites significantly altered in dogs infected with *B. canis* and generated by the GC-MS metabolomics approach. Data are presented as box and whiskers plot (mean ± SD). All metabolites shown demonstrated a statistically significant difference between groups by *t*-test (*p* < 0.05). Blue-control samples, red-disease samples.

Among them, six metabolites were significantly changed. Myo-inositol and methionine were lower, while phenylalanine, stearic acid, phosphoric acid, and isoleucine were higher in dogs infected with *B. canis* (Figure 4).

### 2.5. Principal Component Analysis

**Figure 5:**
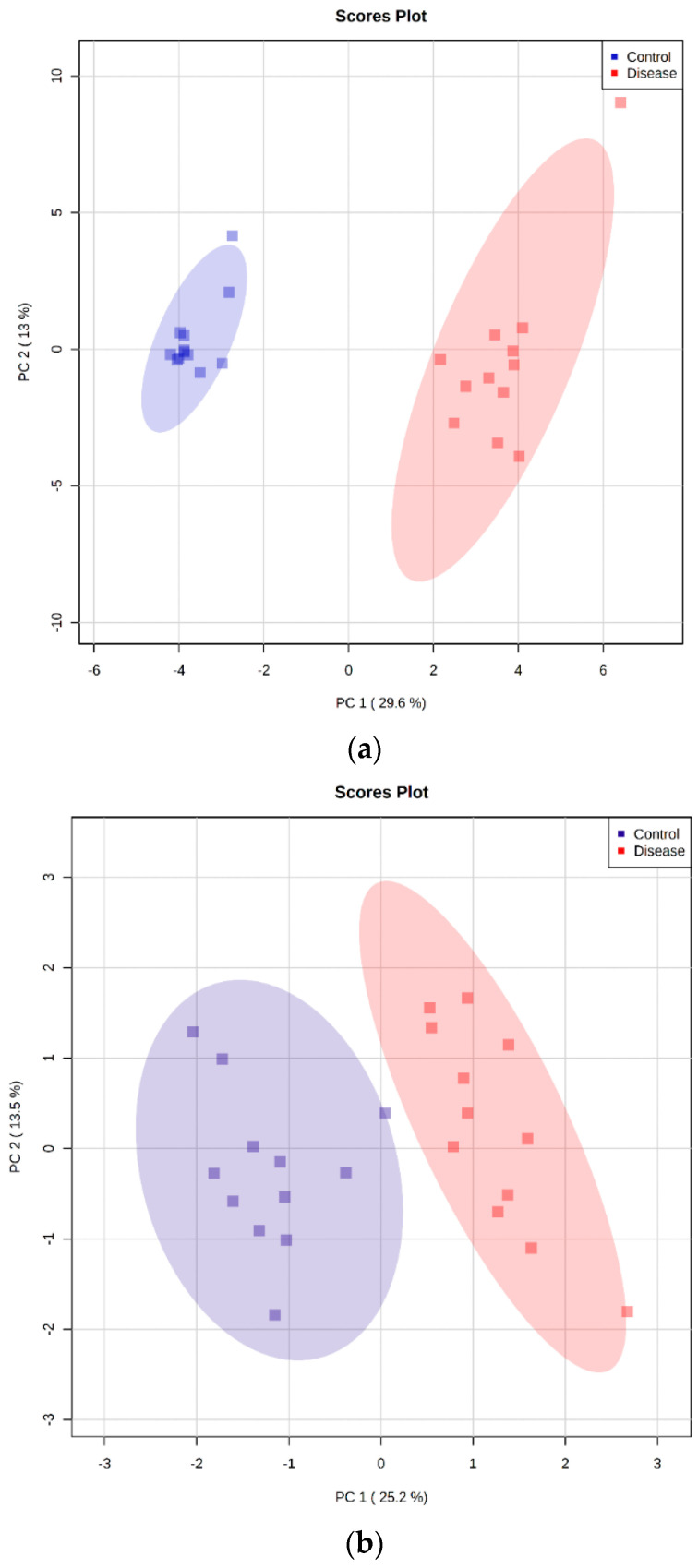

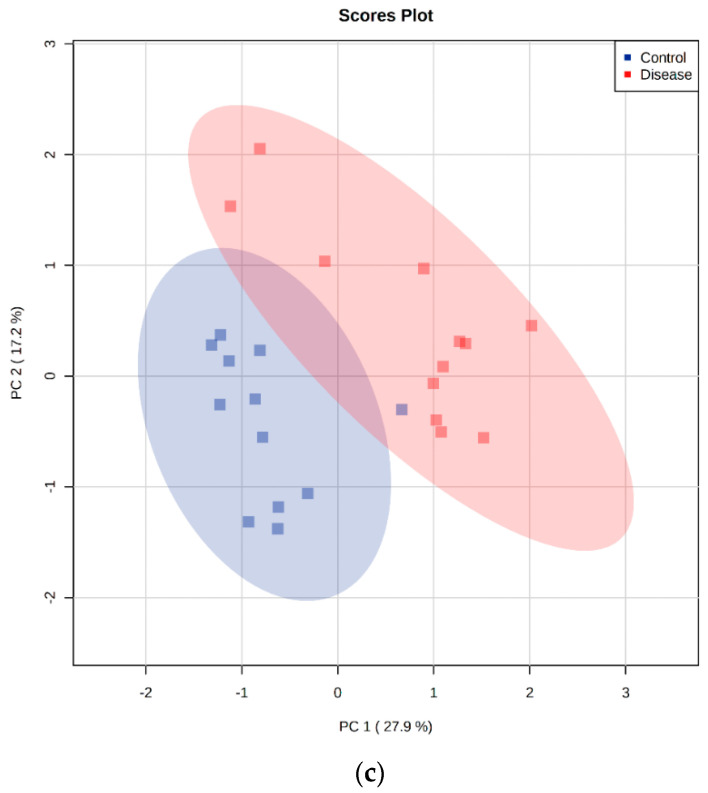
Principal component analysis (PCA) score plots were performed using 24 serum samples of dogs by untargeted LC-MS metabolomics analysis (**a**), targeted LC-MS metabolomics analysis (**b**), and GC-MS metabolomics analysis (**c**). The first two principal components (PCs) explained 426% for the untargeted LC-MS metabolomics, 387% for the targeted LC-MS metabolomics, and 451% of the total variance for the GC-MS metabolomics.

The metabolomics datasets containing 24 samples were analyzed with principal component analysis (PCA) to examine intrinsic variations and identify outliers occurring between the healthy control group and the group with *B. canis* infection. Clustering analysis demonstrated the plotting of samples using two components, principal component 1 (PC1) and principal component 2 (PC2). The PCA plot revealed a clear separation between the analyzed data of the two experimental groups investigated using the untargeted and targeted LC-MS platforms as well as the GC-MS platform (Figure 5). Outliers were not found in the serum of dogs in all three cases.

### 2.6. Hierarchical Clustering Analysis

**Figure 6:**
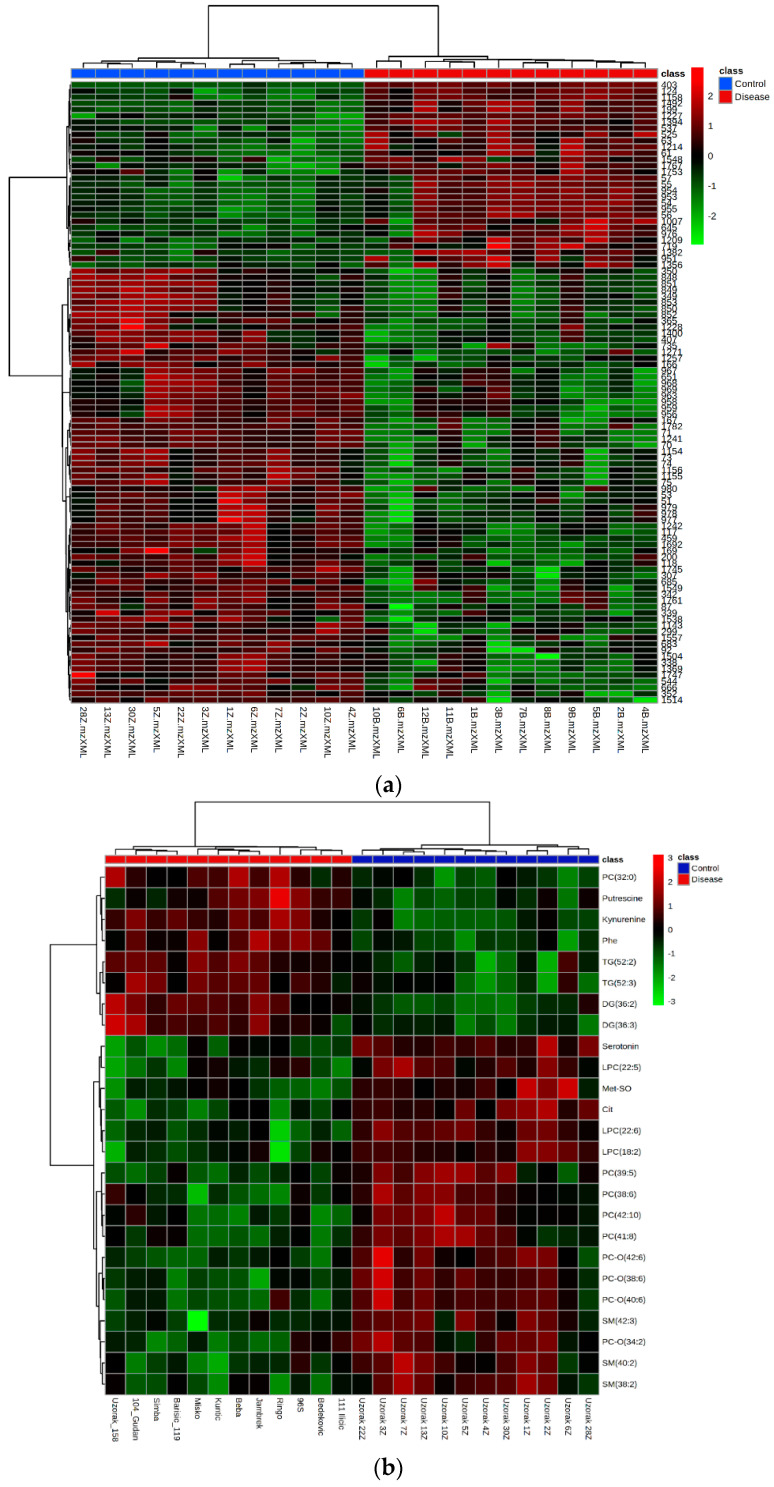

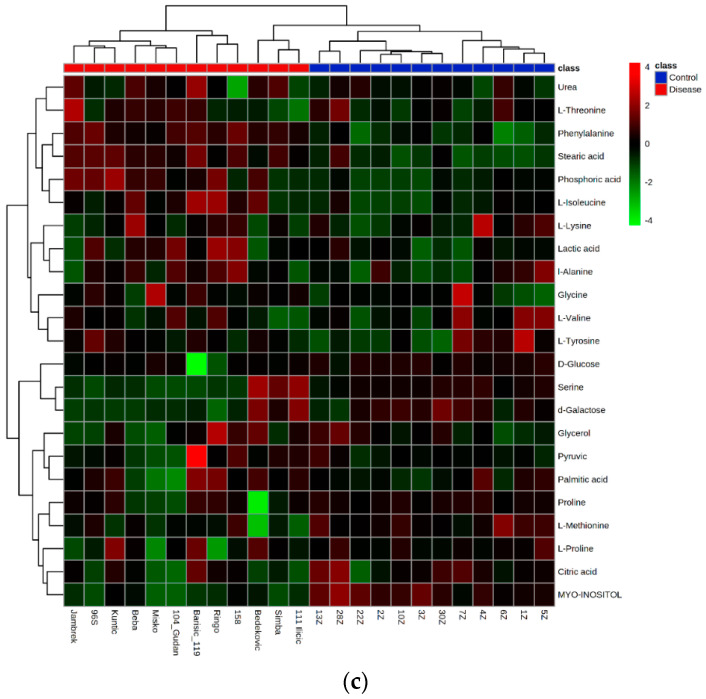
The heat map of the metabolic features in the untargeted LC-MS metabolomics (**a**), targeted LC-MS metabolomics (**b**), and GC-MS metabolomics (**c**). Red color represents the increased level of each metabolite, while blue color represents the decreased level of each metabolite in dogs infected with *B. canis* versus control dogs. Blue panel-control samples, red panel-disease samples.

Heat maps visually represent the differentially abundant metabolic features/metabolites and the alteration in serum metabolome between healthy dogs and dogs infected with *B. canis*. Hierarchical clustering analysis was performed on the peak intensity data obtained from untargeted LC-MS analysis and concentration data from targeted LC-MS analysis and GC-MS analysis. The results that referred to two main clusters were related to the compared samples and metabolic features/metabolites using Euclidean as a distance measure and ward as a clustering algorithm. The metabolites were ranked by *t*-test (*p* < 0.05). Figure 6 shows correct sample group clustering for the untargeted and targeted LC-MS metabolomics and GC-MS metabolomics.

### 2.7. Discriminant Metabolite Identification

**Figure 7:**
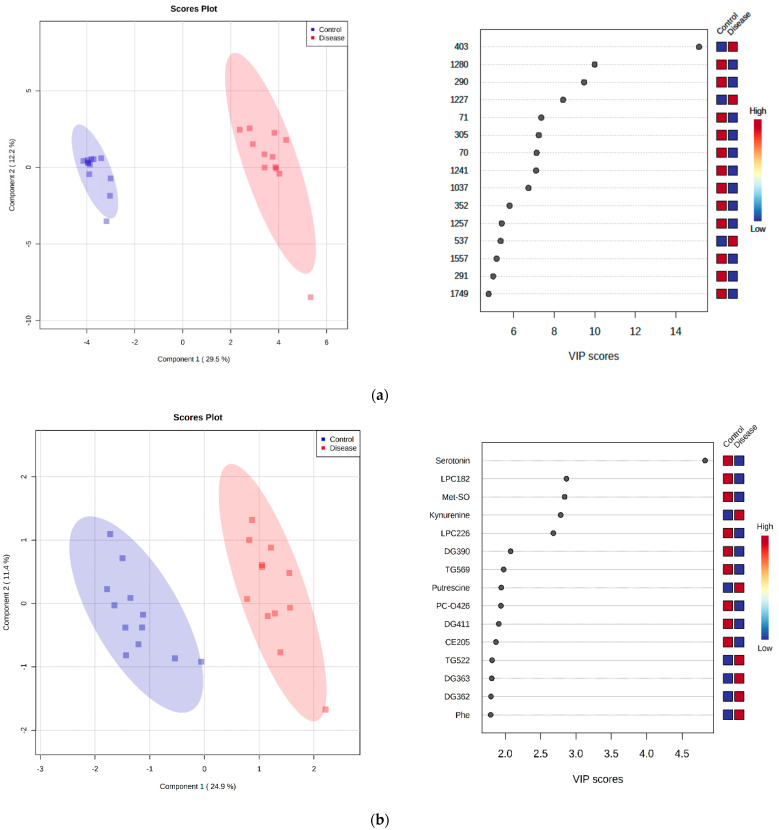

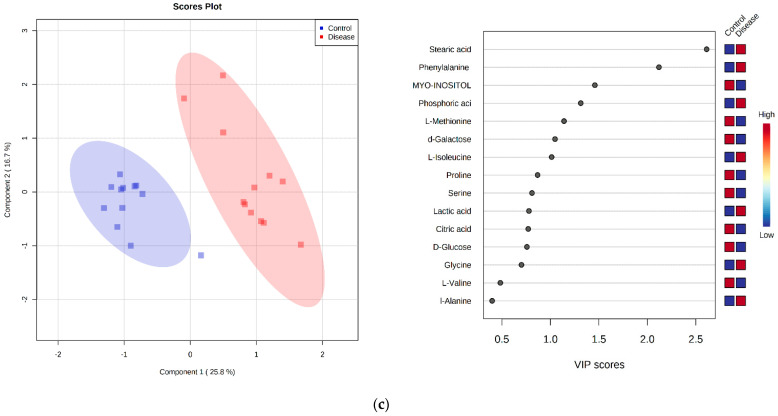
Partial least squares-discriminant analysis (PLS-DA) score plots were performed in 24 analyzed serum samples from control group and group of dogs infected with *B. canis* by untargeted LC-MS metabolomics analysis (**a**), targeted LC-MS metabolomics analysis (**b**), and GC-MS metabolomics analysis (**c**) (left panels). The list of the 15 important compounds/metabolites was identified by PLS-DA according to the variable importance on projection (VIP) score (right panels). The intensity of the colored boxes on the right represents the relative abundance of the corresponding metabolite in each group (blue-control, red-disease).

A partial least squares-discriminant analysis (PLS-DA) was performed to identify metabolites that were the most discriminant between healthy dogs and dogs with babesiosis. The PLS-DA plots represented a clear intergroup separation between the two experimental groups. From the PLS-DA, the differentiation between healthy dogs and dogs with babesiosis occurred along component 1, measured as 29.5% for the untargeted LC-MS metabolomics, 24.9% for the targeted LC-MS metabolomics, and 25.8% for the GC-MS metabolomics, respectively (Figure 7a–c left panels). The validity of PLS-DA was confirmed by cross-validation and showed that the best classifier model comprised five components (R2 = 0.98946, Q2 = 0.96356) for untargeted metabolomics, (R2 = 0.99933, Q2 = 0.86175) for targeted metabolomics, and (R2 = 0.92723, Q2 = 0.8097) for GC-MS metabolomics. The overall VIP scores of the PLS-DA results produced the list of the 15 most important variables contributing to the separation in the PLS-DA plot. These variables are ranked by variable importance in the projection (VIP). The colored boxes on the right indicate the relative concentrations of the corresponding metabolite in each studied group. Some of the important metabolites identified by this separation included the following: inosine (peak 1280, 290), hypoxanthine (peak 70, 1241), and choline phosphate (peak 352) for the untargeted LC-MS metabolomics; serotonin, methionine-sulfoxide (Met-SO), kynurenine, putrescine, and phenylalanine for the targeted LC-MS metabolomics; and stearic acid, phenylalanine, myo-inositol, phosphoric acid, methionine, galactose, and isoleucine for the GC-MS metabolomics (Figure 7a–c right panels).

### 2.8. Pathway Analysis and Enrichment Analysis

**Figure 8:**
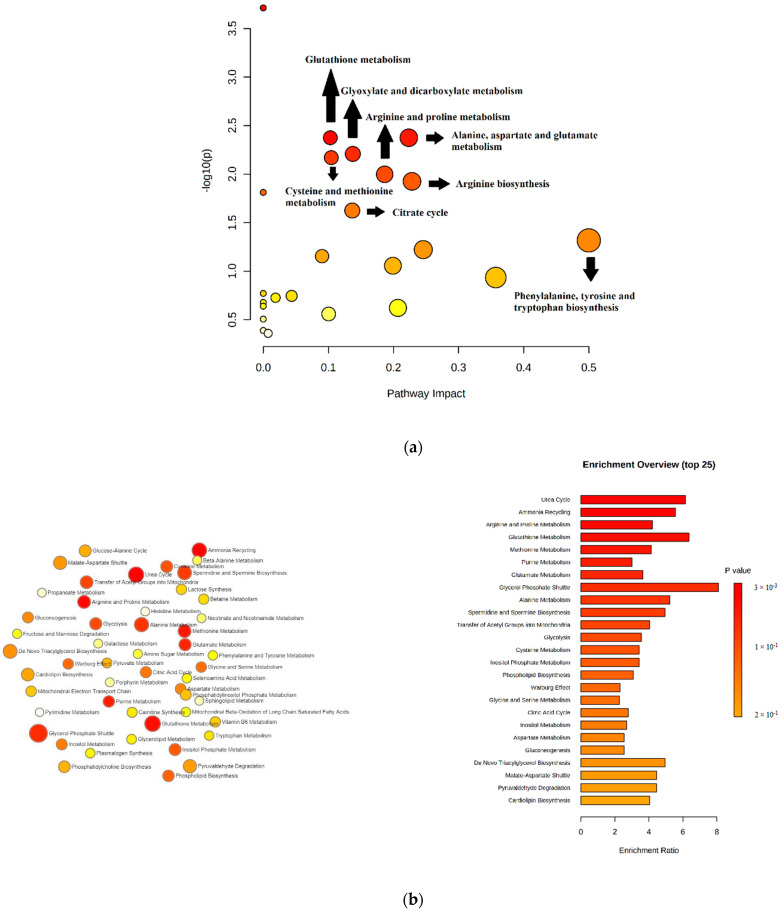
Pathways’ analysis plot of the disturbed metabolic pathways in dogs infected with *B. canis* compared to healthy dogs (**a**). Glutathione metabolism, as well as alanine, aspartate, and glutamate metabolism, glyoxylate and dicarboxylate metabolism, cysteine and methionine metabolism, arginine and proline metabolism, arginine biosynthesis, citrate cycle and phenylalanine, tyrosine, and tryptophan biosynthesis were significant pathways with a *p* value of <0.05 and impact of >0.1. Biological pathways network and summary plot derived from metabolite set enrichment analysis for prediction of pathways associated serum metabolites sets (**b**). The colors and the bar length represent the metabolites with different levels of significance for enrichment analysis.

The significantly identified metabolites (*p*-value < 0.05) obtained by the univariate analysis datasets investigated on the untargeted and targeted LC-MS platform and GC-MS platform were evaluated by MetaboAnalyst v.4.0 [36] for exploring dysregulated metabolic pathways. We performed a pathway analysis using 37 compounds that changed significantly between healthy dogs and dogs infected with *B. canis*. The pathway analysis gave us an overview of metabolites significantly included in the metabolic pathways related to *B. canis*. Pathway analysis resulted in the 24 matched pathways according to *p*-values from the pathway enrichment analysis and pathway impact from pathway topology analysis (Table S3A). Pathways with a *p* value of <0.05 and pathway impact of >0.1 were considered significantly modulated. Overall, the following were the most significantly affected metabolic pathways: glutathione metabolism; alanine, aspartate, and glutamate metabolism; glyoxylate and dicarboxylate metabolism; cysteine and methionine metabolism; arginine and proline metabolism; arginine biosynthesis; citrate cycle; and phenylalanine, tyrosine, and tryptophan biosynthesis (Figure 8a). Metabolite set enrichment analysis (MSEA) combines functionally related metabolites to consider consistent changes between the related metabolites. This analysis identified 25 sets of metabolites (Table S3B). The results of enrichment analysis demonstrated that the most important metabolites participated in the urea cycle, ammonia recycling, arginine and proline metabolism, glutathione metabolism, methionine metabolism, purine metabolism, glutamate metabolism, and glycerol phosphate shuttle (Figure 8b). Table 4 demonstrates identified metabolites included in the significantly altered pathways in canine babesiosis.

## 3. Discussion

To the authors’ knowledge, this is the first report on metabolomics and changes in the composition of serum metabolites in dogs with *B. canis* infection. To obtain a comprehensive metabolite profile and associated biological pathways in dogs with *B. canis* infection, we combined different metabolomics approaches and multiple analytical platforms. Specifically, untargeted and targeted LC-MS-based serum and GC-MS-based serum metabolomics investigations were performed. The combination of three different analytical platforms by applying untargeted and targeted approaches in this investigation resulted in 295 identified metabolites. The Venn diagram confirmed the overlapping of metabolites identified using these three platforms (Figure 1). The LC-MS analysis for untargeted metabolomics detected a total of 147 metabolic features that were significantly changed, with 14 different organic compounds and amino acids matched to the standards. The targeted metabolomics approach on the LC-MS platform identified 68 significant metabolites, while GC-MS analysis identified a total of 6 significantly changed metabolites between groups.

The results of pathway analysis confirmed eight significantly changed metabolic pathways in canine babesiosis (Table 4). According to the pathway analysis, 5-oxoproline, glycine, and putrescine were significant metabolites included in glutathione metabolism. 5-oxoproline is an endogenous molecule obtained from L-glutamate in the gamma–glutamyl cycle. It accumulates in cerebrospinal fluid, blood, and urine of patients affected by some forms of glutathione synthetase (GS) deficiency [37]. The gamma-glutamyl cycle is necessary for the synthesis and breakdown of glutathione (GSH), and a metabolic defect of the gamma–glutamyl cycle leads to glutathione synthetase (GS) deficiency, resulting in an increased level of 5-oxoproline in biological samples. GS deficiency could result in hemolytic anemia, metabolic acidosis, and severe neurological disorders. In our study, glutamate was identified, and the level of 5-oxoproline was increased in dogs with babesiosis. In relation to oxidative stress potentially caused by the elevated concentration of 5-oxoproline [37], the results of Crnogaj et al. (2017) showed a decrease in antioxidant biomarkers (SOD, GPx, and catalase) in dogs with babesiosis [38].

Glycine is a simple amino acid synthesized from serine that consists of an amino and carboxyl group attached to a carbon molecule. This amino acid is essential for neonatal growth and development with a protective activity in different diseases such as ischemic-reperfusion injury [39], oxidative stress [40], endotoxemia [41], and necrotizing enterocolitis [42]. Previously, different studies showed that glycine has anti-inflammatory, immunomodulatory, and cytoprotective activity [43,44], but also that it has a therapeutic potential in inflammatory bowel disease [45,46,47]. The protective effects of glycine are probably due to its direct effect on inflammatory cells such as macrophages to suppress inflammatory cells’ activation [43]. Maybe the same mechanism of protection by glycine is in our samples infected with *B. canis*, due to the acute-phase response of inflammatory during babesiosis. A recent investigation showed that glycine is reduced in the acute phase of malaria [48]. In our study the level of glycine was lower in dogs infected with *B. canis*. The possible reason for the reductions in the level of glycine in dogs with babesiosis could be secondary to parasite uptake and/or host utilization as it is during the acute phase of malaria [49].

A PLS-DA analysis showed that putrescine is one of the most important metabolites for distinguishing infected dogs with *B. canis* from a control group (Figure 7b). Putrescine is a polyamine detected using the LC-MS platform in a targeted metabolomics approach. It can be synthesized by decarboxylation of ornithine [50], an amino acid that was elevated in dogs infected with *B. canis*. The investigation of Rojas-Martinez et al. (2017) confirmed that putrescine is an essential factor for in vitro proliferation of *B. bovis* [51]. Our study confirmed an upregulated level of putrescine in *Babesia* samples, which may be related to the fact that the *Babesia* parasite is using putrescine for proliferation. However, Cook et al. (2007) reported on the role of polyamines in other protozoan parasites such as *Leishmania*, *Trypanosoma*, and *Toxoplasma* [52]. In addition to its role in proliferation, a high concentration of polyamines may also be related to the pathogenesis of malaria [53]. Pathway analysis showed that putrescine is included in the arginine and proline metabolism that is an altered pathway in babesiosis.

In our study, we determined lower levels of serum imidazole-4-acetate, which is involved in histidine metabolism. Imidazole-4-acetate is a naturally occurring compound in the brain and one of the most important imidazole derivatives [54]. Additionally, imidazole-4-acetate is a histidine and histamine metabolite, formed by histidine metabolism without histamine as intermediate or by direct oxidative deamination of histamine. Branco et al. (2018) demonstrated that histamine is a potent inflammatory mediator, and it can promote inflammatory and regulatory responses that contribute to pathological processes [55]. MacGlashan, 2003, showed that histamine is a mediator of inflammation [56]. Therefore, imidazole-4-acetate could be attributed to inflammation processes in babesiosis [56].

Citrate, aspartate, and pyruvate are metabolites included in several of the most significantly affected metabolic pathways in canine babesiosis (Table 4). In our study, citrate was a downregulated metabolite involved in alanine, aspartate, and glutamate metabolism, as well as glyoxylate and dicarboxylate metabolism and the citrate cycle. As an essential metabolic regulator of energy production [57], it is metabolized within the mitochondria from acetyl-CoA and oxaloacetate by citrate synthase [58] and can be exported from mitochondria through citrate carrier (CIC) in the cytoplasm [59]. On the other hand, in the cytoplasm, it is metabolized by ATP–citrate lyase (ACLY) to acetyl-CoA and can be used for fatty acid synthesis [60]. ACLY has been shown to be essential for inflammation; therefore, citrate modulates inflammatory responses in lipopolysaccharide-induced monocytes [61]. Zotta et al. (2020) discussed that citrate might be a critical signal in immunity and inflammation [62]. The downregulated level of citric acid may disrupt cell signaling, interrupt cellular differentiation, and induce apoptosis in dogs with babesiosis due to limited cellular energy supply. A similar effect was found in aplastic anemia [63].

Aspartic acid (aspartate) is a nonessential amino acid that is made from glutamic acid by enzymes using vitamin B6 [64]. It has numerous important biological roles and functions, such as urea formation, pyrimidine synthesis, transport of nicotinamide adenine dinucleotide (NADH) to mitochondria, and generation of alanine and gluconeogenesis [65]. Our study confirmed that aspartic acid is elevated in babesiosis, as in obesity and bladder cancer [66,67]. Interestingly, in an animal model for depression, the level of aspartate is downregulated [68]. Aspartate is involved in several significantly altered metabolic pathways in *B. canis* infection, such as alanine, aspartate, and glutamate metabolism and arginine biosynthesis.

In our study, pyruvate is a key metabolite involved in a network of five different metabolic pathways (alanine, aspartate, and glutamate metabolism, as well as glyoxylate and dicarboxylate metabolism, cysteine and methionine metabolism, arginine and proline metabolism, and the citrate acid cycle). Pyruvate could modulate key regulatory signal pathways in the cytosol and the mitochondrial matrix [69] and protect against hypoxic stress. Additionally, it could help maintain the energetic status that is disrupted during hypoxia. In addition to the above, it has been shown to reduce oxidative stress and protect mitochondrial metabolism [70]. Our results showed that pyruvate is increased in dogs infected with *B. canis* and agree with previous studies where pyruvate is also increased in cases infected with *B. rossi* and *B. bovis* [71,72]. This could indicate a protective role of pyruvate in increased prevalence of oxidative stress, which is one of the characteristics of our disease. Additionally, pyruvate may have a role in hypothermia or the death of animals, as previously suggested [71].

Cordy et al. (2019) suggested that the nonessential amino acids glutamine, proline, glycine, arginine, ornithine, and citrulline were decreased in plasma samples in the acute phase of malaria [48]. In our case, proline, glycine, and citrulline were decreased in dogs infected with *B. canis*, which confirms the fact that *Babesia* infection in dogs shows similarities to malaria. Decreased levels of proline indicate that proline, as a known universal antioxidant in mammalian cells [73], can have a protective role in our infected dogs. 

Phosphoric acid and stearic acid are organic and fatty acids identified on the GC-MS platform by the untargeted metabolomics approach, and according to the PLS-DA (Figure 7c), participate in discriminant metabolite identification. The metabolite phosphoric acid, also known as orthophosphoric acid, has numerous functions in the body. It is an essential constituent of the organism, not only in the bones and teeth, but also in many enzyme systems. Phosphorus has a key role in carbohydrate, fat, and protein metabolism [74,75]. The level of phosphoric acid in patients infected with *B. canis* is increased. In our study, the increased level of phosphoric acid could relate to acute kidney injury as a complication of canine babesiosis. 

Additionally, patients with *B. canis* infection had higher levels of stearic acid. It has also been shown that stearic acid causes hypercoagulability of the blood by activating of factor XII and the aggregation of blood platelets [76]. The former results of Barić Rafaj et al. (2001) confirm the role of stearic acid in babesiosis [77]. Namely, the authors found that during *B. canis* infection, the Hageman factor is activated [77]. Stearic acid is increased in hepatocellular carcinoma as a result of energy requirements and cell membrane synthesis due to aggressive cell proliferation [78]. Moreover, the increase in saturated fatty acids can induce hepatocellular apoptosis [79], but in our case, it could be connected with liver dysfunction in babesiosis.

Biogenic amines, serotonin, and kynurenine are in the top 5 of the 15 important metabolites shown by the highest VIP score values for targeted metabolomics (Figure 7b). It is known that during infection with *B. canis*, the acute-phase response is triggered, as evidenced by the increased fibrinogen, C-reactive protein, and serum amyloid A concentration in the blood. Fibrinogen coats platelets, which results in platelet aggregation and activation of the coagulation system [9]. In addition to the above, degranulation of platelets releases vasoactive molecules such as serotonin [80].

Although serotonin is best known for its role as a neurotransmitter, it plays an important role in mood, appetite, metabolism, vasoconstriction, and platelet function [81,82,83,84]. In our research, the level of serotonin was decreased. The reason for the lower level of serotonin could be the number of platelets. Thus, the hallmark symptom associated with canine babesiosis is thrombocytopenia [85], but at the same time, whole blood serotonin is considered a good estimate of platelet serotonin, which contains 99% of the total serotonin in whole blood [86].

Previous studies showed that increase of kynurenine is related to the impairment of endothelium function and elevated oxidative stress [87]. Its level is also significantly correlated with the presence of markers of vascular endothelium dysfunction, such as thrombomodulin [88], which was found to be increased in dogs with babesiosis, as well as other markers of endothelium activation [89]. Therefore, increased kynurenine levels could be contributed to the endothelial dysfunction present in the babesiosis.

Using an untargeted metabolomics approach by the LC-MS platform, this study confirmed that the levels of inosine and hypoxanthine were decreased in dogs with *B. canis*. These metabolites are in the top 10 of the most significant metabolites distinguishing healthy dogs from dogs with *B. canis* infection according to VIP scores (Figure 7a). Previous research has confirmed that inosine has powerful anti-inflammatory effects in vivo and in vitro by decreasing the production of proinflammatory mediators by the host. Unlike inosine, plasma hypoxanthine is used as a diagnostic tool in the diagnosis of hypoxia-related diseases, and hemolytic disorders [90,91]. Our results showing lower levels of hypoxanthine could be related to the smaller number of red blood cells (RBC) and platelets in *B. canis* infection. Wung and Howell (1980) also mention that special care is needed to avoid leakage of hypoxanthine from RBC and platelets [92].

The combination of two different analytical techniques using untargeted and targeted approaches during our investigation resulted in the identification of phenylalanine as a significant metabolite by all three platforms. The results in this study stated that the metabolite phenylalanine had an increased level in serum samples of dogs with canine babesiosis. According to Neumman et al. (2007), liver injury may potentially be the reason for this increase [93]. Furthermore, the increased level of phenylalanine in the serum of infected dogs may be caused by the damage of phenylalanine-4-hydroxylase activity. Phenylalanine-4-hydroxylase is a hepatic and renal enzyme that converts phenylalanine into tyrosine and is related to immune activation and inflammation [94]. However, Dawiskiba et al. (2014) reported that phenylalanine and isoleucine might be the strongest candidates for biomarkers of inflammatory bowel disease [95]. Our result confirmed that isoleucine was a significantly changed metabolite identified using the GC-MS platform and elevated in the serum of dogs with *B. canis* infection.

Targeted metabolomics by applying FIA-MS analysis detected a total of 58 significantly different compounds classified into the groups of lysophosphatidylcholines, diglycerides, phosphatidylcholines, triglycerides, sphingomyelins, and cholesteryl esters. Christensen et al. (2000) demonstrated that erythrocytes infected with *B. bovis* show increases in phosphatidylcholine, phosphatidic acid, diacylglycerol, and cholesteryl esters compared with uninfected erythrocytes [96]. A similar effect was observed for erythrocytes infected with *B. bigemina*. This increase in phosphatidylcholine in infected erythrocytes may be explained by the lipid biosynthetic activities in the *Babesia* parasite. Our study confirmed that phosphatidylcholine and diacylglycerol are increased in canine babesiosis, and cholesteryl esters are decreased. In our research, triglycerides were increased in serum samples with babesiosis. Khovidhunkit et al. (2004), reported that during inflammation, serum triglyceride levels are increased as metabolic changes lead to an increase in very low-density lipoproteins (VLDL) levels [97]. The increase in VLDL secretion arises as a consequence of adipose tissue lipolysis, suppression of fatty acid oxidation, and increased de novo fatty acid synthesis in the liver [97]. Previous studies demonstrated that sphingomyelin has antioxidant potential [98,99]. It can inhibit lipid oxidation by the formation of an H-bond network within membranes [100]. Coliva et al. (2020) suggested that sphingomyelins might prevent the propagation of lipid peroxidation [101]. According to Crnogaj et al. (2015), *B. canis* infection in dogs is associated with a high concentration of lipid peroxidation [102]. Therefore, sphingomyelins can be involved in the prevention of the propagation of lipid peroxidation in *B. canis* infection in dogs.

Within this study, we developed a comprehensive methodological approach, including different analytical platforms, necessary to study biochemical perturbations during the acute infection with *B. canis*. It revealed a complex network integrating the intermediate metabolism (e.g., citrate cycle) and specific pathways involving amino acids, small peptides, polyamines, and specific classes of lipids. Our results further allow for the translation of these biochemistry features into complex pathological interactions reflecting inflammation, oxidative stress, anemia, hypoxia, etc. Ultimately, the findings of this study provide a valuable and reliable source to expand the available biomarkers of *B. canis* infection.

## 4. Materials and Methods

### 4.1. Ethics Statement

This study was approved on 19 September 2018 by the Committee on Ethics of the University of Zagreb, Faculty of Veterinary Medicine (Permit Number: 640-01/18-17/63; 251-61-20/165-18-01). All participants were admitted to the Clinic for Internal Diseases, Faculty of Veterinary Medicine, University of Zagreb, Croatia, and came from the Zagreb area.

### 4.2. Experimental Design

The metabolomics studies involved two groups of animals (Table S4A–C). Group 1 consisted of 12 dogs naturally infected by the parasite *B. canis* treated in the Clinic for Internal Diseases, Faculty of Veterinary Medicine, University of Zagreb, Croatia. The clinical manifestation of these dogs included fever, lethargy, ticks found by the owner or veterinarian, pale mucus membranes, anemia, jaundice, hemoglobinuria or hematuria, splenomegaly, tachycardia, anorexia, and vomiting. Dogs in this group were of various breeds and sex (aged from 8 months to 10 years, 4 females and 8 males). Group 2 (healthy animals) consisted of 12 dogs of various breeds and sexes (6 females and 6 males, aged from 1 to 12 years). These dogs were considered healthy based on physical examination and hematological and biochemical data, and they were presented at the clinic for routine check-up controls.

The diagnosis of babesiosis was confirmed by demonstration of the parasites within infected erythrocytes in thin blood smears stained with May-Grünwald-Giemsa stain. Polymerase chain reaction (PCR) confirmed the presence of the parasite *Babesia canis* in the blood [3]. One dose (6 mg/kg of body weight) of imidocarb dipropionate (Imizol, Shering–Plough, Newton, NJ, USA) was administered to dogs infected with *B. canis* subcutaneously on the day of admission as initiation of treatment.

### 4.3. Blood Sample Preparation and Analysis

The blood samples were collected from the cephalic vein on the day of admission from all the dogs before subcutaneous administration of imidocarb dipropionate (Imizol) for group 1. The samples were prepared for hematological and biochemical analysis. They were placed in tubes with ethylenediaminetetraacetic acid (EDTA) for hematological analysis and in tubes with no anticoagulant for biochemical analysis. Tubes were centrifuged at 1200× *g*. Blood smears and PCR were performed from samples taken on the day of admission. A portion of the obtained serum was used for routine biochemical analysis and establishing biochemical profiles, while the remainder was stored at −80 °C until used for analysis of metabolites. Hematological data of complete blood count was generated using an automatic hematology analyzer, Horiba ABX (Diagnostics, Montpellier, France), while biochemical profiles were obtained according to standard methods using an automated biochemistry analyzer (Olympus AU 600, Olympus Diagnostica GMBH, Hamburg, Germany).

### 4.4. Untargeted LC-MS Metabolomics Analysis

Serum samples for metabolomics analysis were prepared by protein precipitation, centrifugation, and supernatant filtration. Metabolites were extracted using a chloroform/methanol/water (1:3:1, *v*/*v*/*v*) mixture (chloroform, methanol (Honeywell, Charlotte, NC, USA), and water (Merck, Darmstadt, Germany)). A total of 1000 µL of ice-cold extraction mixture was added to 25 µL of each serum sample and vortexed on a cooled (4 °C) mixer for 5 min. Pooled samples were prepared by mixing 10 µL of each sample (control and disease sample) and subjecting them to the extraction solvent. Matrix blank samples contained the extraction solvent. All samples (serum samples, pooled samples, and matrix blank) were subsequently centrifuged at 13,000× *g* for 5 min at 4 °C. The supernatant (200 µL) was separated into analytical vials and stored at −80 °C until used for UHPLC-MS analysis.

Metabolites were separated using hydrophilic interaction liquid chromatography (HILIC) on a Dionex UltiMate 3000 UHPLC system (Thermo Fisher Scientific, Hemel Hempstead, UK) coupled to a Thermo Orbitrap Q Exactive Plus (Thermo Fisher Scientific; Bremen, Germany). The extracted analyte from the samples was loaded on a ZIC-pHILIC column (150 mm × 4.6 mm, 5 μm column, Merck Sequant, Darmstadt, Germany). The column temperature was maintained at 30 °C, and metabolites were eluted using a gradient composed of a two solvent system, where solvent B was 100% acetonitrile (Honeywell, Charlotte, NC, USA) and solvent A was 20 mM ammonium carbonate (Honeywell, Charlotte, NC, USA) in water. Separation of the metabolites was achieved by using a linear gradient of 80% to 5% of mobile phase B for 15 min, then held at 5% B for 2 min, returned to 80% B for 1 min, and equilibrated for 6 min with a flow rate of 0.3 mL/min. The injection volume was 10 μL in every run, and samples were maintained in the autosampler at 5 °C during analysis. Each sample was run in a randomized order, with a pooled sample run between every 4 samples. The mass spectrometer was operated in full-scan acquisition mode on both positive and negative polarities using electrospray ionization at a mass resolution of 70,000 and a full scan of the *m/z* range of 70–1050. The MS setting was acquired with source voltage of +3.8 kV for positive and −3.8 kV for negative polarities, sheath gas of 40 (arbitrary units), auxiliary gas of 5 (arbitrary units), and capillary temperature of 320 °C. A standard sample containing a mix of 148 reference compounds and metabolites was used for metabolite identification. Quality-control samples containing metabolites extracted from beer and human urine were used in metabolomics analysis for checking signal reproducibility and the quality of the chromatography. A standard mix and quality-control samples were provided by Glasgow Polyomics, College of Medical, Veterinary, and Life Sciences, University of Glasgow, UK.

Raw LC-MS data obtained from each sample were analyzed with the MSconvert tool (ProteoWizard Software Foundation, San Diego, CA, USA) and the Polyomics integrated Metabolomics Pipeline (PiMP) available at http://polyomics.mvls.gla.ac.uk (accessed on 9 March 2021)—using a standard protocol and default parameters [103]. Raw data were converted from the Thermo Scientific ‘RAW’ file format to an ‘mzXML’ file format, centroided, and split into positive and negative polarities using ProteoWizard software [104] and imported into PiMP. A mixture of 148 authentic compounds was run at the beginning and the end of the sample batch to allow for metabolite identification (MSI level 1). Metabolite identification was performed in PiMP, according to the metabolomics standards initiative (MSI) guidelines, by matching mass and retention times of detected peaks with authentic standards, while annotations were made when matching to a metabolite by accurate mass only using metabolite libraries search (e.g., The Human Metabolome Database, HMDB, available at https://hmdb.ca/, (accessed on 5 May 2021)) and/or Kyoto Encyclopedia of Genes and Genomes, KEGG, available at https://www.genome.jp/kegg/, (accessed on 5 May 2021)) integrated within the PiMP standard procedure.

### 4.5. Targeted LC-MS Metabolomics Analysis

Serum metabolites were analyzed using the Absolute IDQ p400 kit (Biocrates Life Science AG, Innsbruck, Austria), a commercially available assay that was originally developed for plasma and allows for the targeted metabolomic analysis of up to 408 metabolites divided into 11 metabolite classes. This kit included amino acids (21), biogenic amines (21), acylcarnitines (55), glycerophospholipids (172 phosphatidylcholines and 24 lysophosphatidylcholines), glycerides (42 triglycerides and 18 diglycerides), hexoses (including glucose), and cholesterol esters (14). Metabolite quantification was based on a combination of liquid chromatography–mass spectrometry (LC-MS) and flow injection analysis–mass spectrometry (FIA-MS). The LC-MS analysis was used to quantify amino acids and biogenic amines, while FIA-MS was used to quantify all other metabolites, such as acylcarnitines, cholesterol esters, glycerophospholipids, glycerides, sphingolipids, and hexoses.

Metabolites were extracted from dog serum according to the manufacturer instructions provided with the kit. Sample preparation was performed on the specific 96-well plate system for protein removal, internal standard normalization, and derivatization. The calibration standards, quality-control samples (QCs), and internal standard mix were diluted to the required concentration. Serum samples and QCs were then centrifuged at 2750× *g* at 4 °C. Briefly, 10 μL of serum sample was pipetted to the center of the filter on the 96-well kit plate containing the internal standards mix. The samples were dried for 30 min using a Vacuum Manifold (Thermo Scientific, Waltham, USA) and then derivatized with 50 μL of 5% derivatization solution of phenyl isothiocyanate (PITC) (Sigma-Aldrich, St. Louis, MO, USA) in water:ethanol:pyridine at a ratio of 1:1:1 (ethanol (Honeywell, Charlotte, USA), pyridine (BDH PROLABO, Lutterworth, UK)). The plate was incubated for 20 min at room temperature and dried again for 60 min using a Vacuum Manifold (Thermo Scientific, Waltham, MO, USA). Metabolites were extracted by addition of 300 μL of 5 mM ammonium acetate (Sigma-Aldrich, St. Louis, MO, USA) solution in methanol and shaking at 450 rpm for 30 min at room temperature. The extracts were collected using a Vacuum Manifold for 2 min into a capture plate for FIA-MS analysis. For LC-MS analysis, a total of 150 μL from the capture plate was transferred and diluted with 150 μL LC-MS-grade water to another plate, while 250 μL of FIA mobile (made by mixing 290 mL MeOH and a 10 mL ampule of Biocrates FIA mobile phase additive, provided with the kit) was added directly to each well of the original capture plate. QCs, such as one blank sample, three zero samples (phosphate-buffered saline (PBS) (BDH PROLABO, Lutterworth, UK)), and three quality-control samples (QC1–3), of which the QC2 samples were injected in five replicates, were also added to the kit plate.

Metabolite extracts were analyzed using a Dionex Ultimate 3000 UHPLC system (Thermo Fisher Scientific, Hemel Hempstead, UK) coupled to a Q-Exactive Plus hybrid quadrupole-Orbitrap mass spectrometer (Thermo Fisher Scientific, Bremen, Germany) using an electrospray ionization source. The samples were loaded on a Thermo p400 HR UHPLC column provided with the kit (available only from Biocrates), and the column temperature was maintained at 50 °C. Metabolites were eluted using a mobile phase A (0.2% formic acid (Sigma-Aldrich, St. Louis, MO, USA)) in H_2_O and mobile phase B (0.2% formic acid in acetonitrile). The injection volume was 5 μL in every run. The total run time was 5.81 min using a gradient of 0% to 95% of mobile phase B over 4 min and a flow rate of 0.8 mL/min. In the FIA-MS analysis, metabolites were eluted using FIA mobile phase at 0.05 mL/min for the first 1.6 min, then the flow rate was increased to 0.2 mL/min for 1.2 min, and then decreased back to 0.05 mL/min for the rest of the program. Instrument analysis was performed in full-scan acquisition mode in both positive and negative modes for LC-MS and FIA-MS, respectively, and all parameters were set according to the guidelines from the Biocrates instructions.

Data were processed according to the manufacturer’s protocol using the Biocrates Met*I*DQ software (Biocrates Life Science AG, Innsbruck, Austria). Quantitation of LC-MS metabolites was performed with XCalibur Quan 4.1 software (Thermo Fisher Scientific, Waltham, MA, USA) based on a 7-point calibration curve and isotope-labelled internal standards for most analytes, unlike the FIA-MS analysis, which used a single-point calibrator with representative internal standards. A total of 3 replicates of blank PBS samples were used to calculate of the limits of detection (LOD).

### 4.6. GC-MS Metabolomics Analysis

Metabolites were extracted using water/methanol/chloroform (1:2.5:1, *v*/*v*/*v*) extraction solution. An aliquot of serum (25 µL) of each sample was extracted using a volume of 250 µL of extraction solution, followed by incubation for 30 min at 37 °C. The samples were then centrifuged at 16,000× *g* for 5 min at 4 °C. A volume of 225 µL of extracts was added to 200 µL of distilled water and shaken at 1200 rpm for 30 min at 37 °C, followed by centrifugation at 16,000× *g* for 5 min at 4°C. The supernatant (225 µL) was separated into an analytical vial and evaporated to dryness using a Speedvac concentrator (Thermo Fisher Scientific, Waltham, MA, USA) for 4 h. A dried aliquot was reconstituted with 40 µL methoxyamine hydrochloride (20 mg/mL) (Sigma-Aldrich, St. Louis, MO, USA) in pyridine. The resultant mixture was mixed via shaking at 1200 rpm for 90 min at 30 °C. Subsequently, a volume of 20 µL of N-methyl-N-trimethylsilytrifluoroacetamide (MSTFA) (Sigma-Aldrich, St. Louis, MI, USA) was added, and the mixture was incubated for 30 min at 37 °C. After derivatization, the mixture was centrifuged at 16,000× *g* for 5 min at 20 °C and stored at −80 ˚C until GC-MS analysis.

The metabolic profiling analysis was carried out on a Shimadzu single quadrupole GCMS-QP2010 gas chromatograph–mass spectrometer (Shimadzu, Kyoto, Japan). Metabolites were separated on a 30 m × 0.25 mm × 0.25 μm BPX-5 capillary column (SGE, Austin, TX, USA), and an aliquot (1 μL) of each derivatized sample was injected splitlessly with a ratio of 1:80. The injector port temperature was held at 250 °C, and high-purity helium was used as the carrier gas at a constant flow rate through the column of 1 mL/min. The total GC run time was 60 min for each sample. The GC temperature was programmed with initial temperature at 60 °C for 2 min, then increased to 330 °C at 15 °C/minute and maintained for 10 min. Full-scan spectra were acquired after a 6 min solvent delay with an MS ion source temperature of 200 °C and interface temperature of 280 °C. The selected mass range was set to 45–600 *m/z* with scan time of 1 s.

Metabolomics data were analyzed using the Shimadzu GCMSsolution software Version 2.53 (Shimadzu, Kyoto, Japan). The retention time correction of peaks was performed based on the retention time of the internal standards peak area using the Automatic Adjustment of Retention Time (AART) function of the Shimadzu GCMSsolution software. The identification of low molecular weight metabolites was carried out using a commercially available GC-MS Metabolite Mass Spectral Database (Shimadzu, Kyoto, Japan), such as the 2.0 NIST library.

### 4.7. Statistical Analyses

Statistical analyses were performed with the online available platform MetaboAnalyst v.4.0 [36]. Untargeted data were log-transformed to improve normality, targeted data were normalized by median, log-transformed, and mean-centered, while GC-MS data were log-transformed and Pareto-scaled prior to principal component analysis (PCA), partial least squares-discriminant analysis (PLS-DA), variable importance on projection (VIP), and heat map clustering. PCA was used as an unsupervised method to profile the distribution of raw data and for analyzing samples without group information to reveal outliers and gain an overview of inner variations in the dataset. A supervised PLS-DA classification method was used to identify the important metabolites. Variable importance in the projection (VIP) plot ranked the metabolites based on their importance in discriminating dogs infected with *B. canis* from healthy dogs. Metabolites with the highest VIP values are the most powerful group discriminators; therefore, metabolites with VIP values of >1 are significant and metabolites with VIP values of >2 are highly significant. Metabolite contents were compared using student *t*-test for both groups of dogs with FDR correction systematically applied across all *t*-tests. Metabolites with a *p*-value of < 0.05 were considered statistically significant.

Pathway analysis and Metabolite Set Enrichment Analysis was performed with MetaboAnalyst v.4.0. (http://www.metaboanalyst.ca, accessed on 14 September 2021) using significant joint metabolites identified by untargeted and targeted metabolomics approach. A hypergeometric test and relative-betweenness centrality were used for the pathway-enrichment analysis and the pathway-topology analysis, while homo sapiens (humans) was selected as the pathway library. The pathway-associated metabolite sets (SMPDB; 99 metabolite sets based on normal human metabolic pathways) was selected as the pathway library for Metabolite Set Enrichment Analysis (MSEA).

## 5. Conclusions

We found that metabolomic profiles of dogs infected with *B. canis* distinguish significantly from healthy dogs. The study combined untargeted and targeted LC-MS and GC-MS metabolomics approaches. The combination of these analytical platforms resulted in the identification of 295 metabolites. Our results suggested that the metabolic changes in canine babesiosis are linked to glutathione metabolism, as well as alanine, aspartate, and glutamate metabolism, glyoxylate and dicarboxylate metabolism, cysteine and methionine metabolism, arginine and proline metabolism, arginine biosynthesis, the citrate cycle, and phenylalanine, tyrosine, and tryptophan biosynthesis. The present study provides a comprehensive overview of the metabolome in dogs infected with *B. canis* and in-depth insight into *B. canis* infection at the molecular level. Our research could help to better understand the pathogenesis of this disease and encourage further research into babesiosis. Metabolomic investigation of serum of dogs with *B. canis* infection provided a new perspective into the metabolic abnormalities identified herein, making our approach a powerful tool to interrogate the host response to a pathogen.

## Figures and Tables

**Table 1:** List of identified and significantly changed metabolites in serum of dogs infected with *B. canis* versus healthy dogs obtained using the untargeted LC-MS metabolomics approach.

Metabolite	Peak ID	Mass	RT(s)	*p*-Value	Log2 (FC)
Inosine	1280	267.0736	591.68	1.74 × 10^−10^	4.62
Hypoxanthine	70	137.0458	567.72	3.00 × 10^−6^	2.95
Choline phosphate	352	184.0734	714.56	8.90 × 10^−5^	2.29
Imidazole-4-acetate	169	127.0502	624.14	6.69 × 10^−4^	1.81
Cystine	407	241.031	771.48	1.59 × 10^−4^	1.54
Citrate	848	191.02	828.92	5.74 × 10^−4^	0.95
Citrulline	73	176.103	764.51	1.94 × 10^−5^	0.91
Methionine	51	150.0584	610.06	1.50 × 10^-4^	0.76
Glycerol-3-phosphate	1657	171.0066	713.52	3.26 × 10^−3^	0.62
Proline	1004	114.0561	659.98	2.94 × 10^−3^	0.58
Pyroglutamic acid	951	128.0354	575.74	3.86 × 10^−4^	−0.70
Phenylalanine	54	166.0863	551.14	9.88 × 10^−7^	−1.13
Pyruvate	1209	87.0088	501.48	3.05 × 10^−5^	−1.15
Kynurenine	199	209.0921	580.41	5.04 × 10^−9^	−1.72

**Table 2:** List of identified and significantly changed metabolites in serum of dogs infected with *B. canis* versus healthy dogs obtained by targeted LC-MS metabolomics approach.

Metabolites	Short Name	*p*-Value	Log2 (FC)	Classification
Serotonin	Serotonin	1.14 × 10^−6^	2.59	Biogenic amines
Methionine sulfoxide	Met-SO	1.39 × 10^−4^	1.79	Biogenic amines
Citrulline	Cit	2.50 × 10^−6^	1.01	Amino acids
Proline	Pro	2.34 × 10^−3^	0.78	Amino acids
Methionine	Met	5.87 × 10^−3^	0.70	Amino acids
Glycine	Gly	1.45 × 10^−2^	0.42	Amino acids
Aspartic acid	Asp	4.02 × 10^−3^	−0.83	Amino acids
Phenylalanine	Phe	2.32 × 10^−6^	−1.06	Amino acids
Putrescine	Putrescine	2.09 × 10^−4^	−1.22	Biogenic amines
Kynurenine	Kynurenine	7.76 × 10^−8^	−1.59	Biogenic amines

**Table 3:** List of all metabolites identified in serum samples of dogs infected with *B. canis* versus healthy dogs obtained by GC-MS metabolomics approach. NA—not available.

Metabolite	Short Name	HMDB ID	Chemical Class
Lactic acid	Lac	00190	Organic acid
Alanine	Ala	00161	Amino acid
Glycine	Gly	00123	Amino acid
Pyruvic acid	Pyruvic acid	00243	Organic acid
3-Hydroxybutyric acid	3-Hydroxybutyric acid	00011	Organic acid
Valine	Val	00883	Amino acid
Urea	Ur	00294	Organic acid
Glycerol	Glycerol	00131	Carbohydrate
Phosphoric acid	Phosphoric acid	NA	Organic acid
Isoleucine	Ile	00172	Amino acid
Proline	Pro	00162	Amino acid
Serine	Ser	00187	Amino acid
Threonine	Thr	00167	Amino acid
Methionine	Met	00696	Amino acid
L-Proline	L-Pro	00162	Amino acid
Glutamic acid	Glu	00148	Organic acid
Phenylalanine	Phe	00159	Amino acid
Isoleucyl-Glutamine	Isoleucyl-Glutamine	28905	Amino acid
Citric acid	Citric acid	00094	Organic acid
Glucose	Glc	00122	Carbohydrate
Lysine	Lys	00182	Amino acid
Tyrosine	Tyr	00158	Amino acid
Galactose	Gal	00143	Carbohydrate
Palmitic acid	Palmitic acid	00220	Fatty acid
Myo-inositol	Myo-inositol	00211	Sugar alcohol
Stearic acid	Stearic acid	00827	Fatty acid

**Table 4:** Identified metabolites correlated to the significantly altered metabolic pathways in dogs infected with *B. canis*.

Metabolic Pathways	Significant Metabolites (Fold Change)	*p*-Value	Pathway Impact
Glutathione metabolism	5-oxoproline (up) Glycine (down) Putrescine (up)	0.0073447	0.10
Alanine, aspartate, and glutamate metabolism	Citrate (down) Aspartate (up) Pyruvate (up)	0.0073447	0.22
Glyoxylate and dicarboxylate metabolism	Pyruvate (up) Glycine (down) Citrate (down)	0.010697	0.14
Cysteine and methionine metabolism	Cystine (down) Methionine (down) pyruvate (up)	0.011653	0.10
Arginine and proline metabolism	Proline (down) Putrescine (up) Pyruvate (up)	0.017166	0.19
Arginine biosynthesis	Citrulline (down) Aspartate (up)	0.017204	0.23
Citrate cycle	Pyruvate (up) Citrate (down)	0.034034	0.14
Phenylalanine, tyrosine, and tryptophan biosynthesis	Phenylalanine (up)	0.048184	0.50

## Data Availability

The mass spectrometry metabolomics raw data are available on request to the correspondence author. All results are presented within the manuscript and/or Supplementary Materials.

## Supplementary Materials

The following are available online at https://www.mdpi.com/article/10.3390/ijms23031575/s1.
